# Role of *NR5A1* Gene Mutations in Disorders of Sex Development: Molecular and Clinical Features

**DOI:** 10.3390/cimb46050274

**Published:** 2024-05-09

**Authors:** Giovanni Luppino, Malgorzata Wasniewska, Roberto Coco, Giorgia Pepe, Letteria Anna Morabito, Alessandra Li Pomi, Domenico Corica, Tommaso Aversa

**Affiliations:** 1 Department of Human Pathology of Adulthood and Childhood, University of Messina, Via Consolare Valeria 1, 98125 Messina, Italy; cocoroberto93@gmail.com (R.C.); giorgia.pepe@unime.it (G.P.); alessandra.lipomi92@gmail.com (A.L.P.); domenico.corica@unime.it (D.C.); tommaso.aversa@unime.it (T.A.); 2 Pediatric Unit, AOU Policlinico G. Martino, Via Consolare Valeria 1, 98125 Messina, Italy; letteria.morabito@gmail.com

**Keywords:** disorder of sex development, fertility preservation, genetic testing, genotype–phenotype correlation, *NR5A1* gene, puberty

## Abstract

Disorders/differences of sex development (DSDs) are defined as broad, heterogenous groups of congenital conditions characterized by atypical development of genetic, gonadal, or phenotypic sex accompanied by abnormal development of internal and/or external genitalia. *NR5A1* gene mutation is one of the principal genetic alterations implicated in causing DSD. This review outlines the role of *NR5A1* gene during the process of gonadal development in humans, provides an overview of the molecular and functional characteristics of *NR5A1* gene, and discusses potential clinical phenotypes and additional organ diseases due to *NR5A1* mutations. *NR5A1* mutations were analyzed in patients with 46,XY DSD and 46,XX DSD both during the neonatal and pubertal periods. Loss of function of the *NR5A1* gene causes several different phenotypes, including some associated with disease in additional organs. Clinical phenotypes may vary, even among patients carrying the same *NR5A1* variant, indicating that there is no specific genotype–phenotype correlation. Genetic tests are crucial diagnostic tools that should be used early in the diagnostic pathway, as early as the neonatal period, when gonadal dysgenesis is the main manifestation of *NR5A1* mutation. *NR5A1* gene mutations could be mainly associated with amenorrhea, ovarian failure, hypogonadism, and infertility during puberty. Fertility preservation techniques should be considered as early as possible.

## 1. Introduction

Disorders/differences of sex development (DSDs) are defined as broad, heterogenous groups of congenital conditions characterized by atypical development of genetic, gonadal, or phenotypic sex accompanied by abnormal development of internal and/or external genitalia [1]. According to the 2006 Chicago Consensus classification, DSDs are divided into three different groups based on karyotype: chromosomal DSDs, 46,XX DSD, and 46,XY DSD. In addition to the proposed classification, DSDs can be part of complex malformations sometimes related to specific genetic abnormalities or multifactorial causes [2]. The chromosomal DSD group is linked to the number and/or structural alterations of the karyotype with abnormality in several of the phases of sex development. The 46,XX and 46,XY DSD groups are caused by single or multiple gene mutations and consequent defects in their corresponding proteins Monogenic causes of DSDs include mutation of single genes implicated in the development of gonadal and genital ducts, gonadal or/and adrenal steroidogenesis and target cell responsiveness. Oligogenic inheritance of different genes implicated in DSDs could partially explain the absence of specific genotype–phenotype correlations. The main genes implicated with DSDs are summarized in Table 1 [3]. Based on these pathological mechanisms, 46,XY DSD can be further subdivided into disorders of sex determination due to abnormal gonadal development, and disorders of sex differentiation characterized by altered production of adrenal and/or testicular hormones or altered peripheral response to steroid hormones [4].

Many genes could be engaged in different molecular processes that lead to DSDs. A significant role in gonadal determination and differentiation is played by the *NR5A1* gene (nuclear receptor subfamily 5 group A member 1), also known as the gene for steroidogenic factor 1 (*SF-1*) and adrenal 4-binding protein. NR5A is a critical factor in adrenal and gonadal development, and in steroidogenesis. The loss of function of the *NR5A1* gene causes different phenotypes in patients with 46,XY DSD and 46,XX DSD and can be associated with additional organ diseases [5].

This review focuses on alterations in the *NR5A1* gene, which cause DSDs through abnormal gonadic development and alteration of steroidogenesis. The authors first briefly describe the process of gonadic development in humans, provide an overview of the molecular and functional characteristics of *NR5A1*, and then discuss the associated clinical phenotypes in detail.

## 2. Overview of Gonadic Development in Humans, Including Interactions of the *NR5A1* Gene and Implicated Effects of Its Mutation

Human gonadal development is a sequential embryological process that begins with chromosomal sex determination (XX or XY) and gonadal ridge formation, leading to the sexual determination and differentiation of the gonads and sex organs. The gonadal development period can be divided into several stages: the indifferent stage, sex determination stage, and sex differentiation stage [6]. In the human embryo, chromosomal sex (XX or XY) is determined from the moment of fertilization, and all embryos possess undifferentiated genital elements in the first six gestational weeks. By the fourth week of gestation, the human gonadal ridge develops on the ventromedial surface of the mesonephros, contingent on several genes implicated as *NR5A1*, *WT1*, *CBX2*, *LHX9*, *EMX2*, and *GATA4*. During embryogenesis, *NR5A1* gene expression has been identified in the urogenital ridge, where it promotes gonadal ridge formation and serves as an early marker of gonadal and adrenal differentiation [6,7,8]. During the fifth week of gestation, bipotential primordial germ cells from the yolk sac migrate to the gonadal ridge, where they differentiate into gonocytes or oogonia cells of the mature gonad. Simultaneously, coelomic epithelial cells of the gonadal ridge initially differentiate into precursors of supporting cells, which further differentiate into Sertoli cells or granulosa cells [9]. Supporting cells are the first cell lines to adopt a sex-specific fate in gonad development, promoting the maturation of the steroidogenic cells, Leydig cells in XY gonads and theca cells in XX gonads, which will produce sex-specific hormones. By the sixth week of gestation, *SRY* (sex-determining region on Y) promotes *SOX9* gene expression, leading to the differentiation of supporting cell precursors into Sertoli cells in the XY gonads [9]. *SOX9* expression is ensured by the binding of SRY and NR5A1 to the TESCO (testis-specific enhancer of SOX9 core) sequence of the *SOX9* gene. Additionally, in vitro studies indicate that the SOX9-NR5A1 complex continuously stimulates *SOX9* and *SRY* expression in pre-Sertoli cells in humans and mice, establishing a male-specific over-regulation mechanism. This underscores the crucial role of *NR5A1* in the development and maintenance of the male differentiation cascade [10,11]. Sertoli cells release paracrine factors such as DHH (desert hedgehog) and PDGF (platelet-derived growth factor) to promote differentiation of fetal Leydig cells [9,12].

Morphological changes, including the initial formation of seminiferous tubules, occur during the seventh to eighth week of gestation. In the fetal testis, Sertoli cells secrete anti-Müllerian hormone (AMH), a critical factor responsible for the regression of Müllerian ducts in male fetuses. By approximately the ninth week of gestation, the Müllerian ducts have almost completely disappeared. The persistence of the Müllerian structure correlates with altered AMH hormone production [8,9,10,11,12,13]. Several transcription factors, including *NR5A1*, *SOX9*, *GATA4* and *WT1*, regulate *AMH* transcription. SOX9 and NR5A1 bind to a conserved site in the promoter region of the *AMH* gene, inducing its expression during early fetal development. NR5A1 plays a crucial role in modulating *AMH* gene expression. NR5A1 interacts with the WT1-KTS complex already bound to the DNA-binding domain of AMH and synergistically up-regulates *AMH* expression [12]. Furthermore, when NR5A1 is linked alone to the promoter of the *AMH* gene, GATA4 can also bind to NR5A1 to upregulate the *AMH* expression, and with further enhancement when two molecules of GATA4 simultaneously interact with NR5A1. If NR5A1 enhances the role of GATA4, FOG-2 can repress GATA4-dependent activation of AMH, particularly in vitro [7,12,14]. Specifically, the *AMH* gene has two different binding sites for the NR5A1 factor. A mutation inactivating the distal SF1 binding site on the AMH promoter causes persistent Müllerian duct syndrome (PMDS) due to ineffective interaction with NR5A1 and GATA4 [15]. Conversely, the repression of the *AMH* expression is mediated by the direct interaction between NR5A1 and DAX1 with the specific promoter region of *AMH* gene. In fact, DAX1 is a transcription factor that inhibits male sexual development [12,13,14,15,16]. In conclusion, *AMH* and its receptor (AMHR2) are regulated by NR5A1.

During the seventh to ninth week of gestation, fetal Leydig cells produce testosterone and insulin-like factor 3 (INSL3), essential for masculinization and development of the male reproductive system, including descent of the testes. Expression of INSL3 is regulated also by *NR5A1* [17]. The *NR5A1* gene also regulates the expression of LHCGR in fetal Leydig cells and promotes steroidogenesis by activating the transcription of steroidogenic enzymes such as CYP11A1 (encoding cytochrome P-450 cholesterol side-chain cleavage), CYP17A1 (encoding 17-alpha-hydroxylase), STAR (encoding steroidogenic acute regulatory protein), and HSD3B2 [18]. Additionally, *NR5A1* regulates metabolic processes such as glycolysis, NADPH synthesis and cholesterogenesis, essential cofactors for steroidogenesis [19]. Testosterone promotes masculinization of the internal genitalia through the differentiation of Wolff’s ducts and, after conversion to dihydro-testosterone by alpha reductase, promotes masculinization of the external genitalia. Both testosterone and dihydro-testosterone act on the same androgen receptor, encoded by the *AR* gene located on the X chromosome [6]. During the development of seminiferous tubules, primordial cells differentiate into gonocytes, with their subsequent proliferation and maturation occurring through paracrine factors secreted by Sertoli and Leydig cells from the 16th week of gestation [20]. In contrast, the differentiation of female genitalia depends on the absence of a Y chromosome, AMH and testosterone. During ovarian development, the *NR5A1* gene plays a tardive role, and its expression starts in the coelomic epithelium at around the seventh week of gestation and increases in the fetal period.

By contrast, during the ovarian cycle in post-menarchal human females, *NR5A1* is expressed in the ovary in the theca interna, in luteinized and non-luteinized granulosa cells and in the corpus luteum [21].

**Figure 1:**
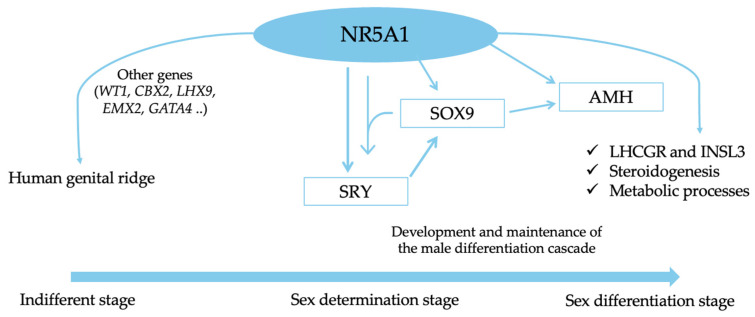
Molecular pathway of *NR5A1* gene during male gonadic development.

In summary, the *NR5A1* gene emerges as a central player in human gonadal development, orchestrating key events essential for sexual differentiation and reproductive function, as summarized in Figure 1 and Table 2. Its intricate regulation highlights its significance and potential implications for DSDs and reproductive health.

## 3. *NR5A1* (Nuclear Receptor Subfamily 5 Group A Member 1)

NR5A1 is a member of the nuclear receptor subfamily, also known as steroidogenic factor 1 (SF-1) and adrenal 4-binding protein (AD4BP). This protein consists of 461 amino-acids encoded by the *NR5A1* gene, located on the long arm of chromosome 9 (9q33.3) [22]. NR5A1 shares several domains with other nuclear receptor subfamily members. In the N-terminal region, it contains the DNA-binding domain (DBD), characterized by two zinc fingers responsible for DNA binding, along with FTZ-F1 box, which stabilizes the interaction with DNA. Functional activation domains (AF-1 and AF-2) further enhance DNA binding and promote transcriptional activity and cofactor interactions. Additional amino acid domains in the C-terminal region are implicated in transcriptional interactions, along with the proline-rich hinge region, which may undergo phosphorylation modifications to enhance stability and transcriptional activity. Another significant region is the ligand binding domain (LBD), a hydrophobic site located between AF-1 and AF-2, filled with phospholipids [22,23]. *NR5A1* is principally expressed in the primary steroidogenic organs, in the adrenal cortex and the gonads [23]. Primordial gonads and the cortex of the cortical adrenal glands share a common embryological origin through the mesonephros. In the adrenal glands, *NR5A1* is expressed in all three zones of the adrenal cortex but is absent in the adrenal medulla, which originates from neural crest cells [24]. In the gonads, *NR5A1* is expressed in the Sertoli and Leydig cells in the testis, and in theca cells, granulosa cells and corpus luteum in the ovary. In these steroidogenic tissues, *NR5A1* primarily regulates cholesterol metabolism, facilitating steroid hormone biosynthesis. In testicular steroidogenic cells, NR5A1 plays a crucial role during gonadal development, as summarized in Table 2, and continues its function throughout pediatric and adult stages. In the ovaries, NR5A1 plays various roles in follicular development, from follicle growth and maturation to the expression of genes such as INHA (encoding inhibin alpha subunit) and CYP19A1, which encodes the aromatase enzyme responsible for estrogen biosynthesis [18,25]. *NR5A1* is also present in non-steroidogenic tissues such as brain, specifically in the ventral medial nucleus (VMN) of the hypothalamus and gonadotropic cells of the pituitary gland. In the VMH, NR5A1 targets genes such as brain-derived neurotrophic factor (BDNF) and N-methyl-D-aspartate receptor (NMDA-R). In pituitary gonadotropic cells, NR5A1 promotes the action of the reproductive axis by encoding gonadotropin-releasing hormone receptor (GnRH-R) and the Beta subunits of luteinizing hormone (LH) and follicle-stimulating hormone (FSH). During brain development, *NR5A1* is expressed in the diencephalon and forming hypothalamus [26]. In addition, *NR5A1* is detected in tissues such as the spleen, skin, and placenta, though its target genes in these tissues remain unidentified [24].

## 4. *NR5A1* Gene Mutation and Disorders/Differences of Sex Development

The *NR5A1* gene plays a crucial role in early gonadal development, testis determination and steroidogenesis. It induces the expression of genes involved in the development and maintenance of the male differentiation process. Mutations in the *NR5A1* gene are considered to be among the main genetic causes of DSDs and are associated with a broad and heterogeneous range of phenotypes [27]. According to the Human Gene Mutation Database, [28] more than 218 *NR5A1* variants of several different types have been characterized.

*NR5A1* gene mutations are some of the most common defects associated with 46,XY gonadal dysgenesis, accounting for up to 10–20% of cases [29]. Analysis of the distribution of the most frequent genetic causes of DSDs in the Ukrainian DSD Registry revealed that the predominant causes are variants in the androgen receptor (AR) gene (32.4%), followed by *NR5A1* gene mutation (14.7%) [30]. Similar results were obtained through gene sequencing of a large international patient cohort [31]: *AR*, *NR5A1* and 5α-reductase type 2 (*SRD5A2*) variants are, respectively, the most frequently identified gene mutations in 187 cases with 46,XY DSD. X-linked AR mutation and autosomal recessive variants of SRD5A2 entail the alteration of androgen synthesis and action, leading to the DSD phenotype in 46,XY patients. In contrast, *NR5A1* mutations affect sex determination and differentiation and cause 46,XX DSD and 46,XY DSD [31,32]. Patients with *NR5A1* mutations may demonstrate an association between DSDs and other organ diseases. The blood, spleen and adrenal gland are the principal organ systems in which anomalies can be associated with *NR5A1* mutations, as *NR5A1* has a significant role in their function and development [33,34].

The SF1next study group conducted an analysis of an international cohort of 197 patients with DSD due to *NR5A1* gene mutations identified through a research network and the I-DSD registry [35]. This retrospective study underlined the heterogeneity of the clinical phenotype and the absence of a specific genotype–phenotype correlation, partly due to possible association with mutations in additional genes related to DSDs. Specifically, the prevalence of *NR5A1* mutation is higher in patients with a 46,XY karyotype (79%) than in those with a 46,XX karyotype (20%). Severe DSD phenotypes predominantly affect patients with a 46,XY karyotype and are also associated with difficulty in interpreting sex at birth and possible abnormal pubertal development. According to the American College of Medical Genetics (ACMG) criteria for predicting the pathogenicity of *NR5A1* variants, severe DSD phenotypes are associated with variants classified as likely benign or benign mutations. Thus, it is not feasible to correlate the clinical phenotype with the predicted pathogenicity of *NR5A1* mutations. In addition, approximately 25% of patients with *NR5A1* mutation present additional gene variants. The association of several gene mutations in the same patients could partially justify the severe clinical phenotype and ambiguity of sex at birth [35]. Consistent with these findings, the SF1next study group identified novel *NR5A1* gene variants that cause altered transcriptional activity and affect nuclear translocation of NR5A1. Genetic analyses did not explain the broad phenotype related to these variants. In addition, additional mutational variants were found in other genes, reinforcing the hypothesis that the broad DSD phenotype associated with *NR5A1* variants may be caused by an oligogenic mechanism [36].

Heterozygous inheritance is the prevalent type of *NR5A1* mutation, and NR5A1 dosage is a relevant determinant of its biological function. Most mutations are localized to the LBD of the *NR5A1* gene, and the protein encoded presents various degrees of alterations that influence the interaction with a DNA molecule [35,37]. Several studies have correlated the clinical phenotype of patients with the genomic site and type of inheritance of *NR5A1* mutations. However, in both cases, a specific correlation has not been highlighted. Additionally, most patients have single-nucleotide substitutions due to missense or nonsense mutations [32]. Missense variants account for 58% of *NR5A1* variants [38]. Meanwhile, some patients present frameshift, insertions, deletions, or complex variants, with no clear correlations between the type of mutation and clinical phenotypes [35,36,37,38].

*NR5A1* variants cause a wide range of clinical phenotypes, from a 46,XY DSD with complete or partial gonadal dysgenesis to 46,XX testicular/ovotesticular DSD (Table 3).

### 4.1. 46,XY DSD Due to NR5A1 Mutations

*NR5A1* gene mutations represent one of the most frequent defects associated with 46,XY gonadal dysgenesis, with about 45–50% of patients with 46,XY DSD having the *NR5A1* mutation [37,38,39,40]. The spectrum of DSDs in 46,XY patients carrying *NR5A1* mutations includes a range from partial to complete gonadal dysgenesis, genital ambiguity, penoscrotal hypospadias, micropenis, cryptorchidism, anorchia, or spermatogenic failure [35,36,37,38,39,40,41,42,43,44,45]. Patients undergoing initial clinical valuation for DSD may present a variable age, from neonatal to adolescent age. The extent of external gonadal alterations and the anatomic location of the gonads could influence the age at which clinical findings are first observed. Diagnosis may even occur during adulthood, as evidenced by a case with normal-appearing female external genitalia who was referred for clinical evaluation at 21 years of age due to primary amenorrhea. However, genetic analysis revealed a 46,XY karyotype with a *NR5A1* mutation (c.77G>A) [40]. Late diagnosis of *NR5A1* mutation could be correlated with the misassignment of sex at birth. Ambiguous genitalia at birth may lead clinicians to assign the patient’s sex to the female gender. In a review of the literature on cases of 46,XY DSD and *NR5A1* mutation, Pedace et al. showed that approximately half of patients were assigned to the female gender, and sex reassignment from female to male was performed in 10% of these cases [39]. The main clinical manifestations at birth are atypical external genitalia with clitoromegaly, labial fusion, phenotypic vaginal opening, or female genitalia. Misinterpretation of these clinical phenotypes as typical of the female sex may delay the first clinical evaluation and, therefore, the age of diagnosis. Conversely, other potential clinical phenotypes may lead to the assignment of male sex [27,39,40]. For instance, ambiguous genitalia characterized by hypospadias, micropenis with severe curvature of phallus and single perineal orifice, bifid scrotum and bilateral cryptorchidism were observed in an Italian patient with 46,XY DSD and *NR5A1* mutation (c.690_691dup CTGCAGCTG) during the neonatal period [39]. This heterozygous variant is characterized by the addition of three amino-acids (Leu-Gln-Leu) in the N-terminal region of the protein. It was also reported in a female patient with a 46,XX karyotype, primary ovarian insufficiency and short stature [27].

In addition, patients with *NR5A1* mutation may present Müllerian structures at birth. Lourenco et al. sequenced the *NR5A1* gene in four families with histories of both 46,XY DSD and 46,XX primary ovarian insufficiency. In three appearing-female patients, external female genitalia with clitoromegaly or genitalia ambiguity were observed, along with Müllerian structures upon instrumental examination. A heterozygous variant (c.1074dupG) of the *NR5A1* gene, causing a frameshift mutation, was detected in a 46,XY patient with clitoral hypertrophy and urogenital sinus at birth. Ultrasound and magnetic resonance imaging (MRI) documented small Müllerian structures in the pelvis and the presence of gonads with testicular structure in the labia majora [41]. Ultrasonography or MRI could be used to document an abnormal gonadal position in different regions, such as at the labioscrotal site, the inguinal region, or the abdominal cavity [39,40,41,42].

Patients born with ambiguous genitalia require prompt clinical and molecular diagnosis. A novel approach for the diagnosis of 46,XY DSD is that updated genetic testing can be performed as the first line of investigation along with urgent molecular diagnosis (electrolytes, glucose, 17OHP). New genetic testing methods (Next-Generation Sequencing (NGS), Whole-Exome Sequencing (WES), and Genomic Copy Number Variation Analysis (GCH array)) enable early diagnosis of genetically based endocrine disorders. Genetic diagnosis allows for precise sex assignment, appropriate management, and accurate prognosis of patients [42,43,44]. In summary, the clinical phenotypic presentation of patients with 46,XY DSD due to *NR5A1* variants are varied. Ambiguous genitalia are the most frequent clinical manifestation, followed by female external genitalia with clitoromegaly, apparent sex reversal compared with karyotype, and male sex with hypospadias [39].

*NR5A1* promotes and regulates steroidogenesis. Mutations in this gene result in variation in basal levels of sex hormones and gonadotropins from the earliest months of life. Köhler et al. documented a low basal testosterone level in all three patients described with 46,XY DSD and *NR5A1* mutation. Low basal testosterone and impaired testosterone response to human chorionic gonadotrophin (hCG) stimulation were observed in a patient with *NR5A1* mutation (c.103-3CO A/WT) born with a micropenis, scrotal hypospadias and bilateral inguinal gonads with the presence of a vaginal rest as a rudimentary Mullerian structure. In this patient, gonadal dysgenesis was detected at birth and confirmed by low inhibin B and AMH levels. Conversely, another patient with *NR5A1* mutation (p.Q107X/WT) and 46,XY DSD (penoscrotal hypospadias and bilateral inguinal gonads) presented a low basal testosterone level with an appropriate response to hCG stimulation at seven days of life. Furthermore, the same patients had low AMH and inhibin B levels in infancy, which indicate progressive gonadal dysgenesis [45]. Although the hormone profile in patients with *NR5A1* mutations can be variable, a high percentage of cases present normal testosterone levels (basal and/or after hCG stimulation test) tested in the first five months of life [35,39].

### 4.2. 46,XX DSD Due to NR5A1 Mutations

The *NR5A1* gene plays a crucial role in ovarian function, as it is involved in follicular growth and maturation. It promotes the expression of *INHA* (encoding inhibin alpha subunit) and the *CYP19A1* gene, which encodes for the aromatase enzyme responsible for estrogen biosynthesis. Additionally, *NR5A1* acts as a regulator of steroidogenesis [19]. In patients with 46,XX DSD due to *NR5A1* mutations, various clinical phenotypes can be observed, ranging from testicular and ovotesticular 46,XX DSD to primary ovarian insufficiency (POI) [35] (see Table 3).

Primary ovarian insufficiency (POI) is characterized by primary or secondary amenorrhea, estrogen deficiency with elevated gonadotropin levels and infertility. Syndromic forms of POI can be caused by genetic mutations or chromosomal abnormalities, such as in Turner syndrome, where striated ovaries (ovarian dysgenesis) and immature oocytes are present. POI may manifest as one of several clinical manifestations in these patients, often emerging after other somatic abnormalities and clinical signs, such as short stature. However, most cases of POI 46,XX with pathological *NR5A1* mutations do not exhibit apparent somatic abnormalities [41,46,47].

Jaillard et al. identified *NR5A1* variants in 2.8% of a cohort of 142 women with ovarian deficiency (POI or diminished ovarian reserve (DOR)) or unexplained infertility. They report, for the first time, a novel variant (p.Val15Met) correlated with POI presenting with primary amenorrhea [47]. Other studies have reported that pathogenic variants in *NR5A1* account for 0.26% to 8% of sporadic POI patients [35,41,48]. Lourenco et al. detected *NR5A1* gene mutations in some members of four families with histories of DSD and in 2 out of 25 cases with sporadic ovarian insufficiency [41]. An Italian female patient with POI and short stature presented a 46,XX karyotype, and genetic analysis revealed an in-frame 9bp heterozygous LBD deletion in the *NR5A1* gene, resulting in altered conformation or function of the protein. Another heterozygous *NR5A1* mutation (p.Pro129Leu) was identified in a patient with clitoral hypertrophy and ovarian insufficiency, detected by elevated FSH levels. These mutations lead to a reduction in the expression of *CYP11A1* and *CYP19A1* [41].

Ambiguous genitalia at birth can most commonly result from congenital adrenal hyperplasia due to 21-hydroxylase deficiency, or from less common conditions. Ovotesticular DSD and testicular DSD in a male with a 46,XX karyotype should be promptly evaluated once the main causes of genital ambiguity are ruled out [49]. Testicular DSD is characterized by the presence of testicular tissue and virilized external genitalia in patients with a 46,XX karyotype. Ovotesticular DSD is characterized by the coexistence of both sex tissue types in the gonads of 46,XX individuals [50]. An important *NR5A1* variant (p.Arg92Trp) can act as a molecular switch in human sex development by downregulating the pro-ovarian Wnt4/β-catenin pathway [32]. This variant of the *NR5A1* gene was detected in six 46,XX patients with testicular DSD and in four cases with ovotesticular DSD. Notably, the p.Arg92Trp variant of *NR5A1* was shared by a patient with 46,XX testicular DSD and his sister, who had a 46,XY karyotype associated with partial gonadal dysgenesis characterized by mild clitoromegaly, labial fusion, and gonads in the inguinal region. In this family, maternal inheritance was identified, although the mother was asymptomatic except for irregular periods throughout her life [32,50,51,52].

### 4.3. The Implications of NR5A1 Mutations on Pubertal Development and Fertility

Pubertal development in patients with a DSD may manifest as normal, delayed, incomplete, absent, or atypical, and the timing and modalities of progression of puberty could be influenced by the specific type of DSD [53]. Alteration in pubertal development has been observed in several young patients with *NR5A1* gene mutation, even though normal initiation of the pubertal process was assessed during their follow-up. In a cohort of 109 individuals with *NR5A1* mutation, 62 cases exhibited normal puberty, with only 12 of these patients presenting with a DSD. In contrast, 42 individuals with a DSD phenotype had abnormal puberty defined as Tanner stage outside the correlated age range, with the presence of pharmacological pubertal induction or absence of menarche at age 15 years [35].

In a cohort of patients with 46,XY DSD due to *NR5A1* mutations, all 10 individuals showed signs of spontaneous pubertal development and virilization. Two of these patients experienced a decrease in testicular volume during puberty, despite their serum testosterone concentrations falling within the normal range for their age and sex [54]. In fact, a patient (46,XY) born with ambiguous genitalia due to *NR5A1* (c.983GOT) mutation had an initial increase in testicular volume (testicular volume: 13–15 mL) during the pubertal phase. However, by the end of puberty, this patient developed an impairment of testicular volume (volume testis: 6 mL) and function characterized by azoospermia and reduction in inhibin B and serum testosterone levels [55]. Similar conditions with abnormal testicular volume during precocious or normal pubertal development could be detected in other DSDs due to several gene mutations (*AR*, *GATA4*) or chromosomal disorders such as Klinefelter syndrome [7,56]. In addition, hypergonadotropic hypogonadism is a possible hormonal phenotype found in patient with *NR5A1* mutation. Faienza et al. [5] described subjects with spontaneous virilization at puberty, abnormal pubertal progression, and evolution to hypergonadotropic hypogonadism. Compensated hypergonadotropic hypogonadism, characterized by elevated gonadotropin levels and normal testosterone levels, was detected in two male patients with DSD due to *NR5A1* mutation. By contrast, two sisters presented with hypergonadotropic hypogonadism during puberty, exhibiting decreased testosterone levels. They were born without perinatal problems and were first observed during puberty for hirsutism, the presence of urogenital sinus, and virilization of external genitalia. Genetic analysis detected a novel *NR5A1* variant (c.248T>A), and psychological evaluations confirmed those patients’ intention to maintain the female sex. Estrogen replacement therapy was initiated after surgical resection of testes, which histologically showed structures composed of foci of Leydig cell and seminiferous tubules containing only Sertoli cells [5].

Serum testosterone levels above the reference range during the early phase of puberty and adolescence could be associated with early pubertal development [44,45,46,47,48,49,50,51,52,53,54,55,56,57]. An increase in testosterone and gonadotropins levels was observed in a singular male patient treated with triptorelin for precocious puberty. This patient was born with testicular dysgenesis characterized by hypospadias, a small genital bud, and cryptorchidism, associated with low levels of AMH and testosterone. Genetic analysis performed during puberty detected a heterozygous *NR5A1* variant (c.207del p.). In addition, at age of 16, the same patient underwent fertility preservation, but azoospermia was also detected with testicular sperm extraction (TESE) [57].

Different hormonal profiles during the first month of life and puberty could be linked to the various effects of the *NR5A1* gene in the Sertoli and Leydig cells during these distinct periods. The absence of Mullerian structures indicates optimal levels of AMH, produced by Sertoli cells, during intrauterine life. However, low levels of AMH during the neonatal period indicate gonadal dysgenesis in the presence of clinical phenotypes of DSD. Inadequate virilization of external genitalia suggests insufficient secretion of testosterone by Leydig cells, confirmed by suboptimal testosterone secretion after a hCG-stimulation test [35,54,58]. Steroidogenesis during fetal life depends on the expression of several genes, such as *NR5A1*, and the coordination of fetal Sertoli and Leydig cells. Consequently, alterations in the *NR5A1* gene can impair testosterone production, also due to Sertoli cell dysfunction [59]. Additionally, fetal and adult Leydig cells may be considered different cell types. Adult Leydig cells are less affected by the action of *NR5A1* products during steroidogenesis due to the compensatory action of *NR5A2*. This phenomenon could partially explain the varying degrees of virilization during the neonatal and pubertal period in patients with *NR5A1* mutation [5,60]. During the pubertal period, Sertoli cell disfunction is shown by a progressive reduction in testicular volume as well as low AMH and inhibin B levels with elevated FSH levels. Clinical signs of virilization during the early phase of puberty, associated with serum testosterone levels within the normal range, suggest preserved function of Leydig cells. However, the progressive reduction in testosterone levels and the increase in LH levels suggest Leydig cell injury during and after the puberal phase [35,56,57,61].

The progressive decline in testicular hormone function could parallel degradation in sperm parameters and testis structure. Various degrees of male infertility could be indicators of testicular dysgenesis associated with endocrine dysfunction and failing testosterone levels with advancing age [3,62]. Bashamboo et al [63] detected *NR5A1* mutation in approximately 4% of a cohort of men with severe unexplained spermatogenic failure. *NR5A1* mutation is associated with several phenotypes such as severe and moderate oligozoospermia, azoospermia or cryptozoospermia [63]. Degradation of testicular structure can be evaluated with a testicular biopsy or histological analysis in patients undergoing gonadectomy. Histological profiles vary with different degrees of compromise of all testicular cells. These can show areas of interstitial fibrosis; atrophic seminiferous tubules with Sertoli cells only, cells showing spermatogenesis arrest; or foci/cluster of hyperplasic Leydig cells with cytoplasmic lipid droplets [5,57,58,59,60,61,62,63]. Lipid accumulation in Leydig cells is the expression of steroidogenic failure induced by *NR5A1* mutation through suppression of STAR, CP450scc and CYP11A1 [64]. If testicular biopsy detects germ cells and functional seminiferous tubules, patients with *NR5A1* mutation and spermatogenic failure should be directed to common (cryopreservation or TESE) or innovative (intracytoplasmic sperm injection (ICSI) or in vitro spermatogenesis technology) fertility preservation techniques as early as possible during or after puberty [61,62,63,64].

In conclusion, male and female patients with *NR5A1* mutations may have conserved pubertal development or abnormalities with several clinical phenotypes from amenorrhea, potential signs of POI, on a spectrum extending to hypergonadotropic hypogonadism. The occurrence of spontaneous virilization at puberty contrasts with the neonatal manifestations, which are indicative of lower testosterone action, and requires careful consideration of sex assignment and the timing and type of possible surgical and hormonal interventions. Moreover, patients with *NR5A1* mutations experience impaired fertility with advancing age, regardless of karyotype. As extensively discussed in the literature [61,62,63,64], in patients with impaired fertility and *NR5A1* gene mutation, fertility preservation techniques should be considered during or after puberty.

### 4.4. NR5A1 Gene Mutations and Additional Anomalies

The *NR5A1* gene is expressed in steroidogenic tissues such as the adrenal cortex, as well as in non-steroidogenic tissues including the brain (specifically the ventromedial nucleus (VMN) of the hypothalamus and gonadotropic cells of the pituitary gland), spleen, skin, and placenta [65,66,67,68,69].

The first patient described with an *NR5A1* gene mutation presented with 46,XY DSD, sex reversal and persistent Müllerian structures, along with primary adrenal failure occurring within the first 2 weeks of life. Genetic analysis detected a de novo heterozygous *NR5A1* mutation (G35E) located in the P-box of the DNA-binding domain (DBD) [65]. Another infant with primary salt-losing adrenal failure, 46,XY DSD, and Müllerian structures was found to have a homozygous *NR5A1* mutation (R92Q) in the A-box region [66]. While adrenal failure is a rare but potentially life-threatening condition in patients with *NR5A1* mutations, its incidence is low within this patient population. In a cohort of 117 patients with primary adrenal failure of unknown etiology, *NR5A1* mutations were detected only in two patients with 46,XY gonadal dysgenesis. Conversely, DAX-1 mutations were found in 58% of patients with adrenal hypoplasia congenita, and in eight cases with hypogonadotropic hypogonadism and a family history suggestive of adrenal failure in males. *NR5A1* mutations causing adrenal insufficiency are rare and are likely associated with significant under-androgenicity and gonadal dysfunction in 46,XY individuals [67]. Recent cohort studies have further emphasized the rarity of adrenal insufficiency in patients with *NR5A1* mutations, with only 5 cases identified among 197 patients [35].

The *NR5A1* gene and its counterpart DAX-1 have opposing roles in gonadal and adrenal development. DAX-1 inhibits male sexual development, while *NR5A1* interacts directly with DAX1 to regulate AMH expression. Adrenal insufficiency is more commonly associated with DAX-1 mutations, leading to primary adrenal failure in infancy or childhood, abnormal puberty with hypogonadotropic hypogonadism, and infertility. Conversely, *NR5A1* mutations result in a broad spectrum of DSDs and abnormalities of puberty, such as hypergonadotropic hypogonadism and infertility [67,68]. While adrenal insufficiency is rare in patients with *NR5A1* mutations, it should be considered and evaluated through adrenocorticotropic hormone (ACTH) testing, especially in patients with monoallelic or biallelic *NR5A1*/*SF-1* variants, as it is most frequently observed in patients with these genotypes [35,67].

The SF1next study group identified associated organ anomalies in patients with *NR5A1* mutations, with anomalies of the spleen, blood system, and central nervous system being more frequent than adrenal anomalies. A specific *NR5A1* variant, p.Arg103Gln, was identified in five patients from different families with spleen anomalies [35]. Cases of polysplenia and asplenia were detected in a family with a heterozygous de novo mutation of *NR5A1* (c.1227C>A) [69]. Additionally, the *NR5A1* gene is expressed in the VMN of the hypothalamus, which plays a central role in appetite regulation in humans. Studies in mice suggest that *NR5A1* mutations may also be implicated in the genetic processes underlying obesity [70].

## 5. Conclusions

*NR5A1* gene mutations are considered to be among the main genetic causes of DSDs and are associated with a broad and heterogeneous range of phenotypes. Clinical phenotypes can vary even among patients carrying the same *NR5A1* variant. Oligogenic inheritance may contribute to the heterogeneity of clinical manifestations, and the variable expressivity of the *NR5A1* mutation could also partially explain the absence of specific genotype/phenotype correlations. New genetic analysis techniques represent crucial diagnostic tools that are increasingly employed early in the diagnostic pathway, as early as the neonatal period, when genital ambiguity may be a suspected manifestation of genetic disease. *NR5A1* gene mutations are also identified during puberty, primarily in girls with amenorrhea and ovarian failure. Hypogonadism and infertility are other clinical phenotypes of patients with DSDs due to *NR5A1* mutation. These patients should be closely followed up, and fertility preservation techniques should be considered during or after puberty.

## Figures and Tables

**Table 1:** Several genes implicated in disorders/differences of sex development.

DSD	Genes
Complete or Partial Gonadal Dysgenesis	*ARX*, *AMH*, *ATRX*, *BMP15*, *CBX2*, *DAX1*, *DHH*, *DMRT1*, *EMX2*, *ESR2*, *FGF9*, *FGFR2*, *FOXL2*, *GATA4*, *HHAT*, *HOXA13*, *MAP3K1*, *NR2F2*, *NR5A1*, *NUP107*, *RSPO1*, *SOX3*, *SOX9*, *SOX10*, *SRY*, *TSPYL1*, *WNT4*, *WT1*, *ZFPM2*, and *ZNRF3*
Defects of Steroidogenesis	*AKR1C2*, *AKR1C4*, *CYB5A*, *CYP11A1*, *CYP11B1*, *CYP17A1*, *CYP19A1*, *CYP21A2*, *DHCR7*, *HSD3B2*, *HSD17B3*, *POR*, *SRD5A2*, and *STAR*
Hormone resistance syndromes	*AMHR*, *AR*, *ESR1*, and *LHCGR*

**Table 2:** Main functions of *NR5A1* gene in the several stages of male gonadic development.

Types of Cells	NR5A1 Functions	Stage of Gonadic Development
Cell lineages of the early human gonad	Formation of the genital ridge	Indifferent stage
Sertoli Cells	Differentiation of cell precursors in Sertoli cells	Sex determination
	Activation the transcription of *SOX9* and *SRY* gene	
	Regulation of AMH and AMHR2 expression	Sex differentiation
Leydig cells	Activation the transcription of steroidogenic genes	Sex differentiation
	Regulation of metabolic process of steroidogenic	
	Increase in the expression INSL3 and LHCGR	

**Table 3:** *NR5A1* gene mutation and main clinical manifestations.

Gene Mutation	Phenotypes
*NR5A1* (9q33.3)	46,XY DSD: – XY gonadal dysgenesis partial or complete – Under-virilized male with atypical external genitalia – Hypospadias – Hypospadias with associated manifestation (micropenis, bilateral anorchia and other) – Spermatogenic failure
	46,XX DSD: – POI (premature ovarian insufficiency) – 46,XX testicular DSD – 46,XX ovotesticular DSD
	Puberty and fertility: – Spontaneous virilization – Normal or atypical pubertal progression – Hypergonadotropic hypogonadism – Amenorrhea and POI (46,XX) – Oligo-azoospermia and spermatogenic failure (46,XY)
	Additional anomalies: – Adrenal failure – Anomalies of spleen, blood system and central nervous system

## References

[1] Cools M., Nordenström A., Robeva R., Hall J., Westerveld P., Flück C., Köhler B., Berra M., Springer A., Schweizer K. (2018). Caring for individuals with a difference of sex development (DSD): A Consensus Statement. Nat. Rev. Endocrinol..

[2] Lee P.A., Houk C.P., Ahmed S.F., Hughes I.A. (2006). Consensus Statement on Management of Intersex Disorders. International Consensus Conference on Intersex. Pediatrics.

[3] Camats N., Flück C.E., Audí L. (2020). Oligogenic Origin of Differences of Sex Development in Humans. Int. J. Mol. Sci..

[4] Bertelloni S., Tyutyusheva N., Valiani M., D’Alberton F., Baldinotti F., Caligo M.A., Baroncelli G.I., Peroni D.G. (2021). Disorders/Differences of Sex Development Presenting in the Newborn with 46,XY Karyotype. Front. Pediatr..

[5] Faienza M.F., Chiarito M., Baldinotti F., Canale D., Savino C., Paradies G., Corica D., Romeo C., Tyutyusheva N., Caligo M.A. (2019). NR5A1 Gene Variants: Variable Phenotypes, New Variants, Different Outcomes. Sex. Dev..

[6] León N.Y., Reyes A.P., Harley V.R. (2019). A clinical algorithm to diagnose differences of sex development. Lancet Diabetes Endocrinol..

[7] Aversa T., Luppino G., Corica D., Pepe G., Valenzise M., Coco R., Li Pomi A., Wasniewska M. (2023). A Rare Case of Precocious Puberty in a Child with a Novel GATA-4 Gene Mutation: Implications for Disorders of Sex Development (DSD) and Review of the Literature. Genes.

[8] Hattori A., Fukami M. (2023). Nuclear Receptor Gene Variants Underlying Disorders/Differences of Sex Development through Abnormal Testicular Development. Biomolecules.

[9] Lundgaard R.M., Jørgensen A. (2022). Deciphering Sex-Specific Differentiation of Human Fetal Gonads: Insight From Experimental Models. Front. Cell Dev. Biol..

[10] Sekido R., Lovell-Badge R. (2008). Sex determination involves synergistic action of SRY and SF1 on a specific *Sox9* enhancer. Nature.

[11] Josso N., Picard J.Y. (2022). Genetics of anti-Müllerian hormone and its signaling pathway. Best. Pract. Res. Clin. Endocrinol. Metab..

[12] Lucas-Herald A.K., Bashamboo A. (2014). Gonadal development. Endocr. Dev..

[13] Josso N., Rey R.A. (2020). What Does AMH Tell Us in Pediatric Disorders of Sex Development?. Front. Endocrinol..

[14] Miyamoto Y., Taniguchi H., Hamel F., Silversides D.W., Viger R.S. (2008). A GATA4/WT1 cooperation regulates transcription of genes required for mammalian sex determination and differentiation. BMC Mol. Biol..

[15] Schteingart H.F., Picard J.Y., Valeri C., Marshall I., Treton D., Di Clemente N., Rey R.A., Josso N. (2019). A mutation inactivating the distal SF1 binding site on the human anti-Müllerian hormone promoter causes persistent Müllerian duct syndrome. Hum. Mol. Genet..

[16] Rey R., Lukas-Croisier C., Lasala C., Bedecarrás P. (2003). AMH/MIS: What we know already about the gene, the protein and its regulation. Mol. Cell Endocrinol..

[17] Tremblay J.J., Robert N.M. (2005). Role of nuclear receptors in INSL3 gene transcription in Leydig cells. Ann. N. Y. Acad. Sci..

[18] Bay K., Virtanen H.E., Hartung S., Ivell R., Main K.M., Skakkebaek N.E., Andersson A.M., Toppari J. (2007). Insulin-like factor 3 levels in cord blood and serum from children: Effects of age, postnatal hypothalamic-pituitary-gonadal axis activation, and cryptorchidism. J. Clin. Endocrinol. Metab..

[19] Morohashi K.I., Inoue M., Baba T. (2020). Coordination of Multiple Cellular Processes by NR5A1/Nr5a1. Endocrinol. Metab..

[20] Culty M. (2009). Gonocytes, the forgotten cells of the germ cell lineage. Birth Defects Res. C Embryo Today.

[21] Takayama K., Sasano H., Fukaya T., Morohashi K., Suzuki T., Tamura M., Costa M.J., Yajima A. (1995). Immunohistochemical localization of Ad4-binding protein with correlation to steroidogenic enzyme expression in cycling human ovaries and sex cord stromal tumors. J. Clin. Endocrinol. Metab..

[22] Parker K.L., Schimmer B.P. (1997). Steroidogenic factor 1: A key determinant of endocrine development and function. Endocr. Rev..

[23] Hoivik E.A., Lewis A.E., Aumo L., Bakke M. (2010). Molecular aspects of steroidogenic factor 1 (SF-1). Mol. Cell. Endocrinol..

[24] Kim A.C., Hammer G.D. (2007). Adrenocortical cells with stem/progenitor cell properties: Recent advances. Mol. Cell Endocrinol..

[25] Hanley N.A., Ikeda Y., Luo X., Parker K.L. (2000). Steroidogenic factor 1 (SF-1) is essential for ovarian development and function. Mol. Cell Endocrinol..

[26] Zhao L., Bakke M., Krimkevich Y., Cushman L.J., Parlow A.F., Camper S.A., Parker K.L. (2001). Steroidogenic factor 1 (SF1) is essential for pituitary gonadotrope function. Development.

[27] Domenice S., Machado A.Z., Ferreira F.M., Ferraz-de-Souza B., Lerario A.M., Lin L., Nishi M.Y., Gomes N.L., da Silva T.E., Silva R.B. (2016). Wide spectrum of NR5A1-related phenotypes in 46,XY and 46,XX individuals. Birth Defects Res. C Embryo Today.

[28] Stenson P.D., Ball E.V., Mort M., Phillips A.D., Shiel J.A., Thomas N.S., Abeysinghe S., Krawczak M., Cooper D.N. (2003). Human gene mutation database (HGMD): 2003 update. Hum. Mutat..

[29] Suntharalingham J.P., Buonocore F., Duncan A.J., Achermann J.C. (2015). DAX-1 (NR0B1) and steroidogenic factor-1 (SF-1, NR5A1) in human disease. Best. Pract. Res. Clin. Endocrinol. Metab..

[30] Globa E., Zelinska N., Shcherbak Y., Bignon-Topalovic J., Bashamboo A., McElreavey K. (2022). Disorders of Sex Development in a Large Ukrainian Cohort: Clinical Diversity and Genetic Findings. Front. Endocrinol..

[31] Tyutyusheva N., Mancini I., Baroncelli G.I., D’Elios S., Peroni D., Meriggiola M.C., Bertelloni S. (2021). Complete androgen insensitivity syndrome: From bench to bed. Int. J. Mol. Sci..

[32] Bashamboo A., Donohoue P.A., Vilain E., Rojo S., Calvel P., Seneviratne S.N., Buonocore F., Barseghyan H., Bingham N., Rosenfeld J.A. (2016). A recurrent p. Arg92Trp variant in steroidogenic factor-1 (NR5A1) can act as a molecular switch in human sex development. Hum. Mol. Genet..

[33] Lucas-Herald A., Bertelloni S., Juul A., Bryce J., Jiang J., Rodie M., Sinnott R., Boroujerdi M., Lindhardt Johansen M., Hiort O. (2016). The long-term outcome of boys with partial androgen insensitivity syndrome and a mutation in the androgen receptor gene. J. Clin. Endocrinol. Metab..

[34] Schimmer B.P., White P.C. (2010). Minireview: Steroidogenic factor 1: Its roles in differentiation, development, and disease. Mol. Endocrinol..

[35] Kouri C., Sommer G., Martinez de Lapiscina I., Elzenaty R.N., Tack L.J.W., Cools M., Ahmed S.F., Flück C.E. (2024). SF1next study group. Clinical and genetic characteristics of a large international cohort of individuals with rare NR5A1/SF-1 variants of sex development. EBioMedicine.

[36] Naamneh E.R., de Lapiscina I.M., Kouri C., Sauter K.S., Sommer G., Castaño L., Flück C.E., SF1next Study Group (2024). Characterization of 35 novel NR5A1/SF-1 variants identified in individuals with atypical sexual development: The SF1next study. J. Clin. Endocrinol. Metab..

[37] Fabbri-Scallet H., de Sousa L.M., Maciel-Guerra A.T., Guerra-Júnior G., de Mello M.P. (2020). Mutation update for the NR5A1 gene involved in DSD and infertility. Hum. Mutat..

[38] Lin L., Philibert P., Ferraz-de-Souza B., Kelberman D., Homfray T., Albanese A., Molini V., Sebire N.J., Einaudi S., Conway G.S. (2007). Heterozygous missense mutations in steroidogenic factor 1 (SF1/Ad4BP, NR5A1) are associated with 46,XY disorders of sex development with normal adrenal function. J. Clin. Endocrinol. Metab..

[39] Pedace L., Laino L., Preziosi N., Valentini M.S., Scommegna S., Rapone A.M., Guarino N., Boscherini B., De Bernardo C., Marrocco G. (2014). Longitudinal hormonal evaluation in a patient with disorder of sexual development, 46,XY karyotype and one NR5A1 mutation. Am. J. Med. Genet. A.

[40] Wang H., Zhang L., Wang N., Zhu H., Han B., Sun F., Yao H., Zhang Q., Zhu W., Cheng T. (2018). Next-generation sequencing reveals genetic landscape in 46, XY disorders of sexual development patients with variable phenotypes. Hum. Genet..

[41] Lourenco D., Brauner R., Lin L., De Perdigo A., Weryha G., Muresan M., Boudjenah R., Guerra-Junior G., Maciel-Guerra A.T., Achermann J.C. (2009). Mutations in NR5A1 associated with ovarian insufficiency. N. Engl. J. Med..

[42] Bertelloni S., Dati E., Baldinotti F., Toschi B., Marrocco G., Sessa M.R., Michelucci A., Simi P., Baroncelli G.I. (2014). NR5A1 gene mutations: Clinical, endocrine and genetic features in two girls with 46, XY disorder of sex development. Horm. Res. Paediatr..

[43] Eggermann T., Elbracht M., Kurth I., Juul A., Johannsen T.H., Netchine I., Mastorakos G., Johannsson G., Musholt T.J., Zenker M. (2020). European Reference Network on Rare Endocrine Conditions (ENDO-ERN. Genetic testing in inherited endocrine disorders: Joint position paper of the European reference network on rare endocrine conditions (Endo-ERN). Orphanet J. Rare Dis..

[44] Zhang D., Wang D., Tong Y., Li M., Meng L., Song Q., Xin Y. (2023). A novel c.64G > T (p.G22C) NR5A1 variant in a Chinese adolescent with 46,XY disorders of sex development: A case report. BMC Pediatr..

[45] Köhler B., Lin L., Mazen I., Cetindag C., Biebermann H., Akkurt I., Rossi R., Hiort O., Grüters A., Achermann J.C. (2009). The spectrum of phenotypes associated with mutations in steroidogenic factor 1 (SF-1, NR5A1, Ad4BP) includes severe penoscrotal hypospadias in 46,XY males without adrenal insufficiency. Eur. J. Endocrinol..

[46] Bashamboo A., McElreavey K. (2010). NR5A1/SF-1 and development and function of the ovary. Ann. Endocrinol..

[47] Jaillard S., Sreenivasan R., Beaumont M., Robevska G., Dubourg C., Knarston I.M., Akloul L., van den Bergen J., Odent S., Croft B. (2020). Analysis of NR5A1 in 142 patients with premature ovarian insufficiency, diminished ovarian reserve, or unexplained infertility. Maturitas.

[48] Jiao X., Qin Y., Li G., Zhao S., You L., Ma J., Simpson J.L., Chen Z.J. (2013). Novel NR5A1 missense mutation in premature ovarian failure: Detection in han chinese indicates causation in different ethnic groups. PLoS ONE.

[49] Baetens D., Stoop H., Peelman F., Todeschini A.L., Rosseel T., Coppieters F., Veitia R.A., Looijenga L.H., De Baere E., Cools M. (2017). NR5A1 is a novel disease gene for 46,XX testicular and ovotesticular disorders of sex development. Genet. Med..

[50] Knarston I.M., Robevska G., van den Bergen J.A., Eggers S., Croft B., Yates J., Hersmus R., Looijenga L.H.J., Cameron F.J., Monhike K. (2019). NR5A1 gene variants repress the ovarian-specific WNT signaling pathway in 46, XX disorders of sex development patients. Hum. Mutat..

[51] Swartz J.M., Ciarlo R., Guo M.H., Abrha A., Weaver B., Diamond D.A., Chan Y.M., Hirschhorn J.N. (2017). A 46,XX Ovotesticular Disorder of Sex Development Likely Caused by a Steroidogenic Factor-1 (NR5A1) Variant. Horm. Res. Paediatr..

[52] Igarashi M., Takasawa K., Hakoda A., Kanno J., Takada S., Miyado M., Baba T., Morohashi K.I., Tajima T., Hata K. (2017). Identical NR5A1 missense mutations in two unrelated 46,XX individuals with testicular tissues. Hum. Mutat..

[53] Nordenström A. (2020). Puberty in individuals with a disorder of sex development. Curr. Opin. Endocr. Metab. Res..

[54] Mönig I., Schneidewind J., Johannsen T.H., Huul A., Werner R., Lünstedt R., Birnbaum W., Marshall L., Wünsch L., Hiort O. (2022). Pubertal development in 46,XY patients with NR5A1 mutations. Endocrine.

[55] Tantawy S., Lin L., Akkurt I., Borck G., Klingmüller D., Hauffa B.P., Krude H., Biebermann H., Achermann J.C., Köhler B. (2012). Testosterone production during puberty in two 46,XY patients with disorders of sex development and novel NR5A1 (SF-1) mutations. Eur. J. Endocrinol..

[56] Rohayem J., Nieschlag E., Zitzmann M., Kliesch S. (2016). Testicular function during puberty and young adulthood in patients with Klinefelter’s syndrome with and without spermatozoa in seminal fluid. Andrology.

[57] Teoli J., Mallet D., Renault L., Gay C.L., Labrune E., Bretones P., Giscard D’Estaing S., Cuzin B., Dijoud F., Roucher-Boulez F. (2023). Case Report: Longitudinal follow-up and testicular sperm extraction in a patient with a pathogenic *NR5A1* (SF-1) frameshift variant: P.(Phe70Ser*fs**5). Front. Endocrinol..

[58] Philibert P., Polak M., Colmenares A., Lortat-Jacob S., Audran F., Poulat F., Sultan C. (2011). Predominant Sertoli cell deficiency in a 46,XY disorders of sex development patient with a new NR5A1/SF-1 mutation transmitted by his unaffected father. Fertil. Steril..

[59] O’Donnell L., Whiley P.A.F., Loveland K.L. (2022). Activin a and sertoli cells: Key to fetal testis steroidogenesis. Front. Endocrinol..

[60] Adachi M., Hasegawa T., Tanaka Y., Asakura Y., Hanakawa J., Muroya K. (2018). Spontaneous virilization around puberty in NR5A1-related 46,XY sex reversal: Additional case and a literature review. Endocr. J..

[61] Grinspon R.P., Bergadá I., Rey R.A. (2020). Male Hypogonadism and disorders of sex development. Front. Endocrinol..

[62] Ciaccio M., Costanzo M., Guercio G., De Dona V., Marino R., Ramirez P.C., Galeano J., Warman D.M., Berensztein E., Saraco N. (2012). Preserved fertility in a patient with a 46,XY disorder of sex development due to a new heterozygous mutation in the NR5A1/SF-1 gene: Evidence of 46,XY and 46,XX gonadal dysgenesis phenotype variability in multiple members of an affected kindred. Horm. Res. Paediatr..

[63] Bashamboo A., Ferraz-de-Souza B., Lourenço D., Lin L., Sebire N.J., Montjean D., Bignon-Topalovic J., Mandelbaum J., Siffroi J.P., Christin-Maitre S. (2010). Human male infertility associated with mutations in NR5A1 encoding steroidogenic factor 1. Am. J. Hum. Genet..

[64] Hatano M., Migita T., Ohishi T., Shima Y., Ogawa Y., Morohashi K.I., Hasegawa Y., Shibasaki F. (2016). SF-1 deficiency causes lipid accumulation in Leydig cells via suppression of STAR and CYP11A1. Endocrine.

[65] Achermann J.C., Ito M., Hindmarsh P.C., Jameson J.L. (1999). A mutation in the gene encoding steroidogenic factor-1 causes XY sex reversal and adrenal failure in humans. Nat. Genet..

[66] Achermann J.C., Ozisik G., Ito M., Orun U.A. (2002). Gonadal determination and adrenal development are regulated by the orphan nuclear receptor, steroidogenic factor-1, in a dose-dependent manner. J. Clin. Endocrinol. Metab..

[67] Lin L., Gu W.X., Ozisik G., To W.S., Owen C.J., Jameson J.L., Achermann J.C. (2006). Analysis of DAX1 (NR0B1) and steroidogenic factor-1 (NR5A1) in children and adults with primary adrenal failure: Ten years’ experience. J. Clin. Endocrinol. Metab..

[68] El-Khairi R., Martinez-Aguayo A., Ferraz-de-Souza B., Lin L., Achermann J.C. (2011). Role of DAX-1 (NR0B1) and steroidogenic factor-1 (NR5A1) in human adrenal function. Endocr. Dev..

[69] Colson C., Aubry E., Cartigny M., Rémy A.A., Franquet H., Leroy X., Kéchid G., Lefèvre C., Besson R., Cools M. (2017). SF1 and spleen development: New heterozygous mutation, literature review and consequences for NR5A1-mutated patient’s management. Clin. Genet..

[70] Majdic G., Young M., Gomez-Sanchez E., Anderson P., Szczepaniak L.S., Dobbins R.L., McGarry J.D., Parker K.L. (2002). Knockout mice lacking steroidogenic factor 1 are a novel genetic model of hypothalamic obesity. Endocrinology.

